# Viral-mediated expression of desmin mutants to create mouse models of myofibrillar myopathy

**DOI:** 10.1186/2044-5040-3-4

**Published:** 2013-02-20

**Authors:** Pierre Joanne, Oussama Chourbagi, Christophe Hourdé, Arnaud Ferry, Gillian Butler-Browne, Patrick Vicart, Julie Dumonceaux, Onnik Agbulut

**Affiliations:** 1 Université Paris Diderot, Sorbonne Paris Cité, CNRS EAC4413, Unit of Functional and Adaptive Biology, Laboratory of Stress and Pathologies of the Cytoskeleton, 75013, Paris, France; 2 Department of Aging, Stress and Inflammation, Université Pierre et Marie Curie-Paris 6, Sorbonne Universités, 75005, Paris, France; 3 Université Pierre et Marie Curie-Paris 6, Sorbonne Universités, UMR S794, INSERM U974, CNRS UMR7215, Institut de Myologie, 75013, Paris, France; 4 Université Paris Descartes, Sorbonne Paris Cité, 75006, Paris, France

**Keywords:** Myofibrillar myopathy, Intermediate filament, Desmin, AAV vectors, Phenotypic heterogeneity, Desminopathy

## Abstract

**Background:**

The clinical features of myofibrillar myopathies display a wide phenotypic heterogeneity. To this date, no studies have evaluated this parameter due to the absence of pertinent animal models. By studying two mutants of desmin, which induce subtle phenotypic differences in patients, we address this issue using an animal model based on the use of adeno-associated virus (AAV) vectors carrying mutated desmin cDNA.

**Methods:**

After preparation of the vectors, they were injected directly into the tibialis anterior muscles of C57BL/6 mice to allow expression of wild-type (WT) or mutated (R406W or E413K) desmin. Measurements of maximal force were carried out on the muscle *in situ* and then the injected muscles were analyzed to determine the structural consequences of the desmin mutations on muscle structure (microscopic observations, histology and immunohistochemistry).

**Results:**

Injection of AAV carrying WT desmin results in the expression of exogenous desmin in 98% of the muscle fibers without any pathological or functional perturbations. Exogenous WT and endogenous desmin are co-localized and no differences were observed compared to non-injected muscle. Expression of desmin mutants in mouse muscles induce morphological changes of muscle fibers (irregular shape and size) and the appearance of desmin accumulations around the nuclei (for R406W) or in subsarcolemmal regions of fibers (for E413K). These accumulations seem to occur and disrupt the Z-line, and a strong regeneration was observed in muscle expressing the R406W desmin, which is not the case for E413K. Moreover, both mutants of desmin studied here induce a decrease in muscle force generation capacity.

**Conclusions:**

In this study we show that AAV-mediated expression of desmin mutants in mouse muscles recapitulate the aggregation features, the decrease in contractile function and the morphological changes observed in patients with myofibrillar myopathy. More importantly, our results suggest that the R406W desmin mutant induces a robust muscle regeneration, which is not the case for the E413K mutant. This difference could help to explain the phenotypic differences observed in patients. Our results highlight the heterogeneous pathogenic mechanisms between different desmin mutants and open the way for new advances in the study of myofibrillar myopathies.

## Background

Myofibrillar myopathy (MFM) [OMIM:601419] refers to a group of genetically heterogeneous chronic neuromuscular disorders. MFMs are mainly caused by mutations in the desmin gene [1], while other forms of MFM are caused by mutations in alpha-B-crystallin [2], myotilin [3], ZASP [4], filamin C [5] or BAG3 [6] genes. Rigid spine syndrome caused by mutation in the SEPN1 gene is also considered as a MFM [7]. The morphological changes described in MFM result from disturbance of the sarcomeric Z-line and the myofibrils, followed by abnormal aggregation of proteins predominantly involved in the structure of the Z-line. The age of disease onset can vary from early adolescence to the 60s. The disease is very heterogeneous manifesting in some cases as a relentlessly progressive skeletal myopathy with no signs of cardiac involvement [8,9]. In other cases cardiomyopathy is a prominent [1] or even exclusive [10] feature. Respiratory insufficiency may also be a major manifestation and cause of death [11].

In 1998, Goldfarb and his co-workers [1] established that a mutation in the desmin gene was involved in MFM. Since then, more than 50 different mutations responsible for MFM symptoms have been identified in the desmin gene. Desmin is the major specific intermediate filament protein of skeletal, cardiac and smooth muscles. It forms a three-dimensional scaffold around the myofibrillar Z-disc, and connects the contractile apparatus and other structural elements of the cell, that is, the subsarcolemmal cytoskeleton, the nuclei and other organelles [12,13]. It also links the myofibrils laterally by connecting the Z-discs, and is particularly abundant at the myotendinous and neuromuscular junctions of skeletal muscle and in the intercalated disks of cardiomyocytes [14]. Thus, desmin filaments are essential to maintain cellular integrity, for the transmission of force and the mechanochemical signaling of muscle cells [15-17].

In the present study, we examined two missense desmin mutations, R406W and E413K. A patient with a R406W mutation in desmin was first described by Park *et al*. in 2000 [18]. In 2004, three new cases were described and compared [19]. From this study it became apparent that patients with the R406W mutation have many common features. These four patients were of different nationalities and no other member of their families was affected by the disease (sporadic *de novo* mode of inheritance). The first signs of the disease appeared at around 20 years of age and were characterized by severe cardiac involvement (cardiac arrhythmia and complete atrioventricular block resulting in pacemaker implantation). The disease developed with increasing muscle weakness of the lower limbs. This weakness then extended to the muscles of the upper limbs and the neck. The final outcome of the disease is usually very severe with a high risk of heart failure. The E413K mutation was first described by Pruszczyk *et al*. in 2007, as a disease primarily affecting cardiac function with an autosomal dominant mode of inheritance [20]. Three cases from the same family were described. The first symptoms of heart disease developed later than those of the R406W mutation (age of onset at 30 years versus 20 years). The first signs of muscle weakness appeared about 15 years after the first cardiac symptoms and were restricted to the lower limbs. Therefore, from these reports it would appear that the R406W mutation is more pathogenic than the E413K mutation, since the clinical symptoms develop later and the skeletal muscles are less affected. However, the molecular mechanism underlying these differences in the clinical phenotypes cannot be studied with the cellular models that are currently available, since both desmin mutations R406W and E413K present the same features. Both are unable to form filamentous networks on their own, but are able to integrate into pre-existing desmin networks and form aggregates, which are concentrated around the nuclei and throughout the cytoplasm [21]. Several teams, including ours, have demonstrated that R406W and E413K mutations, located in the terminal consensus motif, alter in a similar manner the dimer interaction, which impairs the filament formation [20-22]. These observations clearly demonstrate that neither *in silico* nor cellular models are able to explain the molecular mechanisms which result in the phenotypic heterogeneity observed in patients carrying these mutations.

To try to address this issue, several transgenic mouse models expressing different mutations of desmin have been obtained [23-25]. However, the different promoters and strains used make the comparison quite difficult. Moreover, conventional techniques of transgenesis do not seem compatible to analyze the large number of mutations which have been identified within the desmin gene. We therefore chose to use a simpler strategy to study, in the same genetic background, different dominant mutations of desmin. In order to develop these models, we produced an adeno-associated virus (AAV) vector that allows the expression of a mutant desmin in muscles. Intramuscular injection of this virus allows the localized transgenic expression of desmin mutants in C57BL/6 mice. Different analyses were carried out first on the muscle *in situ* (measurement of muscular force) and then the injected muscles were analyzed to determine the structural consequences of the desmin mutations on muscle structure (microscopic observations, histology and immunohistochemistry).

It should be noted that studies on MFM are inherently limited because of the small number of patients and very limited access to biopsy. In addition, this strategy of creating an *in vivo* model appears to be very interesting because of its ease and rapidity of obtaining, and the low cost associated with its implementation. Using this model we aim to reproduce *in vivo* the phenotypic variability observed in patients in order to better understand the mechanisms behind these variations.

## Methods

### Plasmids production

The full-length human desmin cDNAs were cloned into pSMD2 plasmid (cytomegalovirus (CMV) promoter and human β-globin pA) using Xho I restriction site. To distinguish the desmin transgene from the endogenous form, a c-Myc tag was introduced at the 5^′^ end of the desmin cDNA. It should be noted that the presence of c-Myc at the N-terminal of desmin does not disturb filament assembly or cellular localization [21]. R406W and E413K desmin mutations were introduced into the pSMD2 plasmid by site-directed mutagenesis using the Stratagene QuikChange kit (Agilent Technologies, Massy, France). *Escherichia coli* Stbl2 strain (Life Technologies, Saint Aubin, France) was transformed with the different plasmids according to the manufacturer’s instructions for amplification. All plasmids were purified using the endotoxin-free PureYield Plasmid Maxiprep System (Promega, Lyon, France), and then verified by restriction enzyme digestion and sequencing (Eurofins MWF Operon, Ebersberg, Germany).

### Production, purification and titration of the AAV vectors

Pseudotyped AAV-2/1 vectors were generated in human embryonic kidney 293 cells by the triple transfection method described by Rivière *et al*., with minor modifications [26]. The productions were realized with pXX6 adenovirus helper plasmid coding the adenoviral sequences essential for AAV production, the pRep-Cap plasmid coding the AAV-1 capsid and the pSMD2-DesWT, pSMD2-DesR406W or pSMD2-DesE413K plasmid coding the different desmin transgenes. After triple transfection of the different plasmids in 293 cells, cells were harvested and submitted to three freeze-thaw cycles using a 37°C water bath and dry ice cooled ethanol. The lysate was incubated with Benzonase (50 U/ml; Sigma-Aldrich, Saint-Quentin Fallavier, France) for 30 minutes at 37°C. It was then clarified by centrifugation at 6,500 rpm for 20 minutes and passed though a 0.45 μm filter. The lysate was layered over the top of an iodixanol gradient solution (from 15% to 60%; Sigma-Aldrich) and centrifuged for 90 minutes at 59,000 rpm. The virus was isolated between the 40% and 60% iodixanol solutions, washed with PBS-MK (PBS, MgCl_2_ 1 mM, KCl 2.5 mM) solution and concentrated on Amicon Ultra-15 centrifugal filter (Millipore, Molsheim, France). The final viral preparations were stored in PBS solution at -80°C. The AAV titer was determined by real-time PCR (TaqMan, Life Technologies) using CMV promoter specific primers (forward: 5^′^-CTACGCCCATTTGCGTCAA-3^′^; reverse: 5^′^-GCACCAAAATCAACGGGAC-3^′^; probe: 5^′^-CAAAATGTCGTAACAACTCCGCCCC-3^′^).

### Intramuscular delivery of AAV vectors

All procedures have been approved by our institutional Ethics Committee, and conducted according to the French and European laws, directives and regulations on animal care (European Commission Directive 86/609/EEC). Our animal facility is fully licensed by the French competent authorities and has animal welfare insurance. For intramuscular injection, 35 15-week-old female C57BL/6 mice were randomized into four groups: PBS (n = 6), AAV-DesWT (n = 11), AAV-DesR406W (n = 10) and AAV-DesE413K (n = 8). Animals were anesthetized by intraperitoneal injection of pentobarbital sodium (60 mg/kg). Each tibialis anterior muscle was injected with 50 μl of either AAV-2/1-Des (10^11^ viral genomes/muscle) or sterile PBS solution as a control.

### Muscle force measurements

Four weeks after injection, mice were anesthetized (pentobarbital sodium, 60 mg/kg) and the limbs were fixed with clamps. The distal tendon of the tibialis anterior muscle was attached to a dual-mode lever arm system that measures muscle isometric force (300C; Aurora Scientific, Ontario, Canada). Great care was taken to ensure that the blood and nerve supply remained intact during surgery. Active force measurements were performed as described previously [27-29]. The sciatic nerve was crushed proximally and stimulated distally by a bipolar silver electrode using supramaximal square wave pulses of 0.1 ms duration. All isometric measurements were made at L0 (muscle length at which maximal force was obtained during the twitch). Force production in response to tetanic stimulation were successively recorded (pulse frequency from 25, 50 and 100 to 143 Hz, 500 ms) and at least 1 minute was allowed between each contraction. The absolute maximal force was normalized to the muscle mass to determine specific maximal force.

At the end of the experiments, the animals were euthanized with an overdose of pentobarbital. After contractile measurements, the muscles were dissected, weighed and frozen in isopentane pre-cooled in liquid nitrogen or fixed in paraformaldehyde for further analysis. Force measurements were made in PBS treated (n = 12) or WT (n = 22), R406W (n = 20) and E413K (n = 15) desmin expressing tibialis anterior muscles 1 month after AAV vectors injection.

### Histological staining

Transversal frozen sections of 10 μm thickness were prepared from all of the muscles. Hematoxylin and eosin staining was used to examine general morphology of the muscles. The muscle mitochondrial distribution and localization was examined using succinate dehydrogenase (SDH) staining. The extent of fibrosis was assessed by Sirius red staining and was expressed as the fibrosis index (ratio of the area of the fibrosis to the area of fibers). All staining methods were carried out as described by Dubowitz [30]. Images were taken with a microscope (Leica Microsystems, Nanterre, France) equipped with a digital camera (QImaging, Surrey, Canada). For fibrosis analysis, nine digital images per muscle (n = 5/group) were processed with ImageJ software (National Institutes of Health, Bethesda, MD, USA) [31].

### Immunofluorescence

Transversal (morphometric analysis) and longitudinal (striation pattern analysis) frozen sections of 8 μm thickness were cut using a microtome (Leica Microsystems) for immunostaining. The sections were incubated with blocking solution (bovine serum albumin, 5%) for 1 hour and then incubated for 30 minutes with goat anti-mouse immunoglobulin G (IgG) Fab fragment (1:100; Jackson ImmunoResearch Europe, Newmarket, UK). Following a PBS wash, the sections were incubated for 90 minutes with primary antibodies against perlecan (1:400, rat monoclonal; Millipore), c-Myc (1:1000, rabbit polyclonal; Sigma-Aldrich), α-actinin (1:100, mouse monoclonal; Sigma-Aldrich) or myosin heavy chain (MHC) isoform neonatal (1:100, rabbit polyclonal) [32]. After washing in PBS, sections were incubated for 1 hour with secondary antibodies (Alexa Fluor; Life Technologies) or phalloidin-TRITC labeled (Sigma-Aldrich). After washing in PBS, slides were finally mounted in Vectashield with DAPI H-1200 (Vector Laboratories, Peterborough, UK). Images were captured using a motorized confocal laser scanning microscope (LSM 700; Carl Zeiss SAS, Le Pecq, France). Morphometric analyses were made using the ImageJ [31] software and a homemade macro. The smallest diameter (minimum Feret diameter) of all the muscle fibers of the whole muscle section was measured. The pattern of striations was analyzed on longitudinal muscle sections using the Plot Profile function of ImageJ [31] software.

### Electron microscopy

Electron microscopy was carried out as described previously [33]. Briefly, the calf muscles of mice were fixed in 2.5% glutaraldehyde buffered in 0.1 M cacodylate at pH 7.4. After 1 hour, the tibialis anterior muscle was dissected and separated in three by a short-axis section and then fixed overnight at 4°C in the same fixative. After washing, the specimens were post-fixed for 1 hour with 1% osmium tetroxide solution, dehydrated and embedded in epoxy resin. Ultrathin sections (70 nm) were cut with an ultramicrotome (Leica UC6; Leica Microsystems) and stained for 15 minutes with 4% uranyl acetate and for 2 minutes with Reynolds’ lead citrate, before observation at 80 kV with a transmission electron microscope (TEM) Philips Tecnai 12 BioTWIN (Philips, Amsterdam, Netherlands) equipped with an Olympus KeenView CCD camera (Shinjuku, Tokyo, Japan).

### Western blot

Immunoblotting was carried out on extracts of muscles snap frozen in liquid nitrogen immediately after dissection. Frozen muscles were placed into an ice-cold homogenization buffer containing: 50 mM Tris (pH 7.6), 250 mM NaCl, 3 mM ethylenediaminetetraacetic acid (EDTA), 3 mM ethylene glycol tetraacetic acid (EGTA), 0.5% NP40, 2 mM dithiothreitol, 10 mM sodium orthovanadate, 10 mM NaF, 10 mM glycerophosphate and 2% of protease inhibitor cocktail (Sigma-Aldrich). Samples were minced with scissors and then homogenized using plastic pestles, incubated for 30 minutes on ice, sonicated three times for 5 seconds with 30 second intervals on ice, and then centrifuged at 12,000 g for 30 minutes at 4°C. Protein concentration was measured using the Bradford method with bovine serum albumin as a standard. Equal amounts of protein extracts (25 μg) were separated by SDS-PAGE before electrophoretic transfer onto a nitrocellulose membrane (Amersham Hybond-ECL; GE Healthcare, Vélizy-Villacoublay, France). Western blot analysis was carried out using anti-c-Myc antibody (1:1,000, mouse monoclonal; Santa Cruz Biotechnology, Heidelberg, Germany) and anti-pan-actin antibody (1:10,000, mouse monoclonal; Millipore). Antibody reacting bands were visualized with peroxidase-conjugated secondary antibodies (Thermo-Fisher Scientific, Brebières, France) and a chemiluminescent detection system (ECL-Plus; GE Healthcare).

### Statistical analysis

Groups were statistically compared using analysis of variance (ANOVA). If necessary, post-hoc analysis was performed using Tukey’s Honestly Significant Difference (HSD) test. For groups that did not pass tests of normality (Shapiro-Wilk) and equal variance (Bartlett), non-parametric tests were used (Kruskal-Wallis) and multiple comparison were driven using the Dunn procedure and multivariate normal distribution in R software. For statistical analysis of fiber size distribution, Kolmogorov-Smirnov test was used. Values are means ±SEM.

## Results

### Morphological modifications of muscles expressing mutated desmin recapitulate MFM

**Figure 1:**
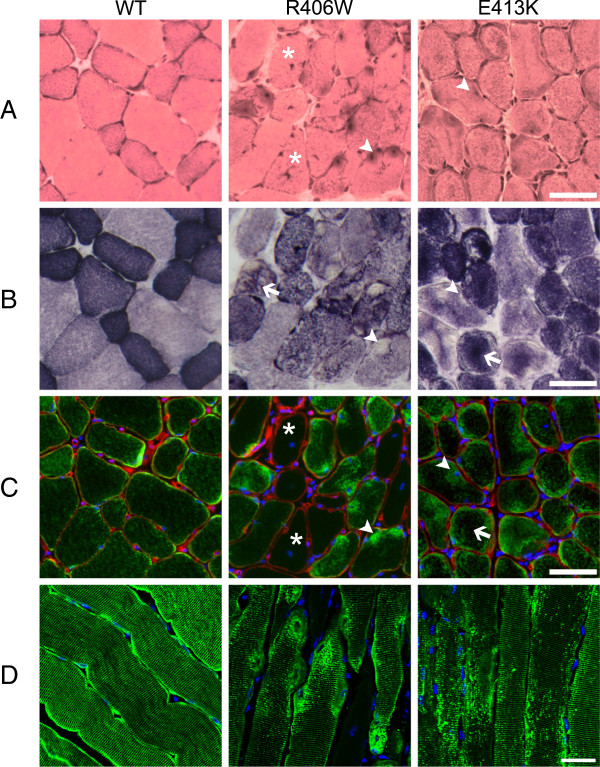
**Morphological perturbations induced by desmin mutants in tibialis anterior muscles.** (**A**) Hematoxylin and eosin staining, (**B**) succinate dehydrogenase (SDH) staining, (**C**) immunostaining against c-Myc (green) and perlecan (red), and (**D**) immunostaining against c-Myc (green); on serial transversal (**A**, **B** and **C**) and longitudinal (**D**) sections of tibialis anterior muscles expressing WT, R406W or E413K desmin. All analyses were performed 1 month after intramuscular injection of AAV vectors. Note that expression of WT desmin does not seem to modify neither muscle morphology (**A** to **B**) nor desmin localization (**C** to **D**), whereas expression of R406W and E413K desmin mutants induces accumulation of the desmin in the perinuclear region (see arrowheads). Arrows in (**B**) illustrate abnormal accumulation of mitochondria observed in both R406W and E413K expressed tibialis anterior muscles. Arrow in (**C**) illustrates a weaker accumulation of desmin in the case of E413K desmin. Asterisks indicate centronucleated muscle fibers. Nuclei (blue) were stained using DAPI (**C** to **D**). Scale bars = 30 μm. AAV, adeno-associated virus; DAPI, 4′,6-diamidino-2-phenylindole; SDH, succinate dehydrogenase; WT, wild-type.

Since the diagnosis of MFM is most often made by using the results obtained from histological stains of muscle sections, we first conducted a series of histological stains of mouse muscles expressing WT or mutated (R406W or E413K) desmin 1 month after injection of the different AAV constructions (AAV-DesWT, AAV-DesR406W and AAV-DesE413K) in the tibialis anterior muscle of C57BL/6 mice (Figure 1A). Normal myofibers with a polygonal shape, peripheral nuclei, intact sarcolemma, non-fragmented sarcoplasm and homogeneous fiber size distribution revealed that overexpression of WT desmin (Figure 1A, WT) had no effect on muscle structure, which was identical to an untreated muscle. On the contrary, expression of the R406W mutant of desmin induced the appearance of darkly stained deposits located mainly in the perinuclear regions of the muscle fiber (see arrowhead in Figure 1A, R406W). We also saw myofibers with central nuclei indicating that the muscle fiber is in the process of regeneration (see asterisks in Figure 1A, R406W) or the nuclei are moving from a peripheral to a central location. Expression of E413K mutant of desmin provoked a similar phenotype but with more blurred dark deposits irradiating from both the subsarcolemmal and perinuclear regions (see arrowhead in Figure 1A, E413K) of the muscle fibers. In addition, many muscle fibers were irregular in shape in muscles expressing the mutated desmin.

In a second step, we performed SDH staining on serial sections to explore the distribution of muscle mitochondria (Figure 1B). Muscles treated with the virus expressing WT desmin showed a mosaic of fibers with a relatively dense purple appearance in oxidative fibers and scattered purple speckles in the nonoxidative fibers. These observations are in perfect accordance to a normal muscle. In contrast, the muscles expressing the R406W mutant showed a localized absence of staining in perinuclear regions (see arrowhead in Figure 1B, R406W) that correspond to the accumulation of dark material revealed by hematoxylin and eosin staining (Figure 1A). An irregular staining of some fibers and in some cases a strong accumulation of staining in the center of fibers (see arrow in Figure 1B, R406W) were also observed. The muscles expressing the E413K desmin mutant also presented many fibers with this abnormal internal architecture characterized by a central dark staining, indicating a modified internal repartition of mitochondria (see arrow in Figure 1B, E413K). In addition, we also observed a decrease in the staining of subsarcolemmal and perinuclear regions (see arrowhead in Figure 1B, E413K), which also corresponded to the accumulation revealed by hematoxylin and eosin staining (Figure 1A).

### Aberrant aggregation of desmin

Next, we performed a series of immunostaining to detect the expression and localization of desmin (Figure 1C,D). To distinguish the desmin transgene from endogenous desmin, a c-Myc tag was introduced at the 5^′^ end of the desmin cDNA. On transversal sections desmin appeared homogeneously distributed throughout the cytoplasm (Figure 1C, WT). On longitudinal sections it was also correctly and repeatedly aligned like sarcomeres (Figure 1D, WT). It should be noted that immunostaining against desmin on AAV-DesWT treated tibialis anterior showed no differences compared to untreated control muscles (data not shown). Furthermore, we observed that there were also no observable differences between the immunostaining against c-Myc and that against desmin in WT expressing tibialis anterior muscle (data not shown), which confirmed perfect integration of exogenous desmin into the normal desmin network.

As seen in Figure 1C,D, compared to immunostaining against c-Myc in WT expressing muscles, we showed significant differences in muscles expressing the R406W and E413K desmin mutants. In these muscles the mutant desmin was strongly accumulated around the nuclei for the R406W desmin (see arrowhead, Figure 1C, R406W). For desmin E413K we observed a much weaker labeling in the center of the fibers and desmin seems to be pushed towards the subsarcolemmal regions (see arrow, Figure 1C, E413K). This observation corresponded well with the central accumulation of staining seen on SDH staining. We also observed, although to a lesser extent than for R406W, accumulation in perinuclear regions of the fibers (see arrowhead, Figure 1C, E413K). These features were also observed in the longitudinal sections where the R406W desmin seemed to strongly accumulate around the nuclei while E413K desmin formed numerous dots occupying the entire width of the fibers (Figure 1D). Moreover, the alignment of the sarcomeres seemed to be disturbed in areas where R406W or E413K desmin were accumulated (Figure 1D). A closer analysis of the Z-lines (α-actinin immunostaining) and thin filaments (phalloidin staining) demonstrated a very clear and localized perturbation of the alignment at the zones of accumulation of desmin (Additional file 1: Figure S1). As seen in Additional file 1: Figure S1, c-Myc was co-localized with α-actinin in both WT and mutated desmin expressing muscles; whereas actin, the major component of the I-band, was not localized with c-Myc. It should be noted that the alignment of Z-lines did not seem to be disrupted at the sites where desmin was not drastically accumulated, as seen in Figure 1D.

### Aberrant muscle regeneration and impaired distribution of muscle fiber size

As shown in Figure 1, many muscle fibers expressing R406W mutant are centronucleated (see asterisks in Figure 1A,C, R406W) suggesting that there is an important muscle regeneration. If this phenomenon really occurs, the absence of c-Myc immunostaining in these centronucleated fibers could be explained by the fact that AAV has never been described to transduce satellite cells.

**Figure 2:**
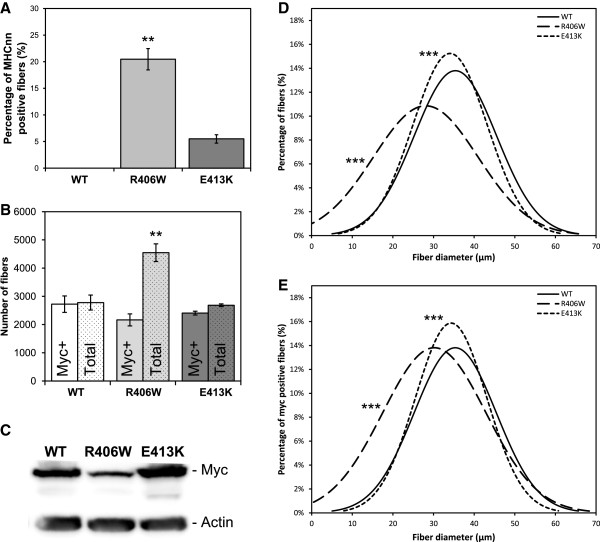
**Regeneration, hyperplasia and decrease in overall fiber size of R406W desmin expressing muscles compared to E413K expressing muscles.** (**A**) Percentage of neonatal myosin heavy chain (MHC) expressing muscle fibers, (**B**) total number (dots filled bars) and c-Myc expressing (empty bar) muscle fibers, (**C**) Western blot quantification of c-Myc, (**D**) size distribution of total and (**E**) c-Myc expressing muscle fibers; were examined in WT (n = 3), R406W (n = 3) and E413K (n = 3) desmin expressing tibialis anterior muscles 1 month after AAV vectors injection. For all morphometric analysis, all muscle fibers in whole muscle sections were analyzed. Note the strong increase in regeneration of tibialis anterior expressing R406W desmin compared to the slight increase with E413K desmin (**A**). Note also hyperplasia of R406W desmin expressing muscle demonstrated after counting the exogenous desmin expressing (Myc-positive, empty bars) and the total number of fibers (dots filled bars) (**B**). To compare size distribution of total (**D**) and exogenous desmin expressing (Myc-positive) (**E**) muscle fibers of WT, R406W or E413K desmin muscles, the Gaussian curve was plotted and grouped. Note the overall decrease in the size of both total and exogenous desmin expressing muscle fibers in R406W muscles, and, to a much lesser extent, the decrease in the size of fibers expressing desmin E413K. Asterisks indicate a significant difference compared to the WT (*P ≤0.05, **P ≤0.01, ***P ≤0.001). AAV, adeno-associated virus; MHC, myosin heavy chain; WT, wild-type.

To confirm that there is an increase of regeneration in muscles expressing mutant desmin, we measured the number of neonatal MHC-positive fibers reported to the total number of fibers from tibialis anterior sections (Figure 2A). For muscles expressing WT desmin, we were not able to detect any neonatal MHC-positive fibers. In the case of muscles expressing R406W or E413K desmin there was, however, an increasing number of fibers expressing this marker (Figure 2A). This ratio was larger and significantly different from WT in the case of R406W desmin (20.5%, *P* <0.01) compared to E413K desmin (5.5%, *P* >0.05).

To confirm that all of these modifications were not due to a difference in the expression levels of exogenous desmin, we performed a Western blot with total protein extract from AAV-treated muscles. We detected a similar amount of exogenous desmin between WT and E413K expressing muscles, whereas in R406W expressing muscles we observed a decrease in the amount of exogenous desmin (Figure 2C). We also counted and determined the c-Myc expression of each muscle fiber of the entire muscle section. Interestingly, after having counted the number of c-Myc-positive muscle fibers for each muscle (Figure 2B), we observed that there was no significant (*P* = 0.252) difference between the muscles expressing WT (2721±291 fibers) and those expressing the R406W desmin (2163±212 fibers) or E413K (2404±68 fibers). In contrast, compared to the WT (2779±265 fibers), the total number of fibers increased significantly in the case of muscles expressing R406W desmin mutant (4546±313 fibers, *P* = 0.0036), which was not the case for muscles expressing E413K (2684±48 fibers, *P* >0.05). This increase in the total number of muscle fibers in R406W expressing muscles may also explain the relative decrease in the amount of exogenous desmin seen on Western blot because newly generated muscle fibers cannot express exogenous desmin.

As expected for a regenerating muscle, the overall distribution of the size of all fibers analyzed from a cross section of tibialis anterior (Figure 2D) treated with AAV-DesR406W is strongly shifted to the smaller sizes (-20.6% of average size compared to WT desmin expressing fibers). We also observed this phenomenon, but to a lesser extent, with the muscles treated with AAV-DesE413K (-3.7%). Focusing this analysis on the fibers expressing only exogenous desmin, we observed very similar results (-14.6% for R406W desmin expressing fibers and -2.9% for E413K desmin expressing fibers, respectively). This observation emphasizes that muscle regeneration is not the sole mechanism responsible for the impaired distribution of muscle fiber size observed in the muscles expressing R406W (Figure 2E).

### Ultrastructural analysis indicates perturbations at Z-lines

**Figure 3:**
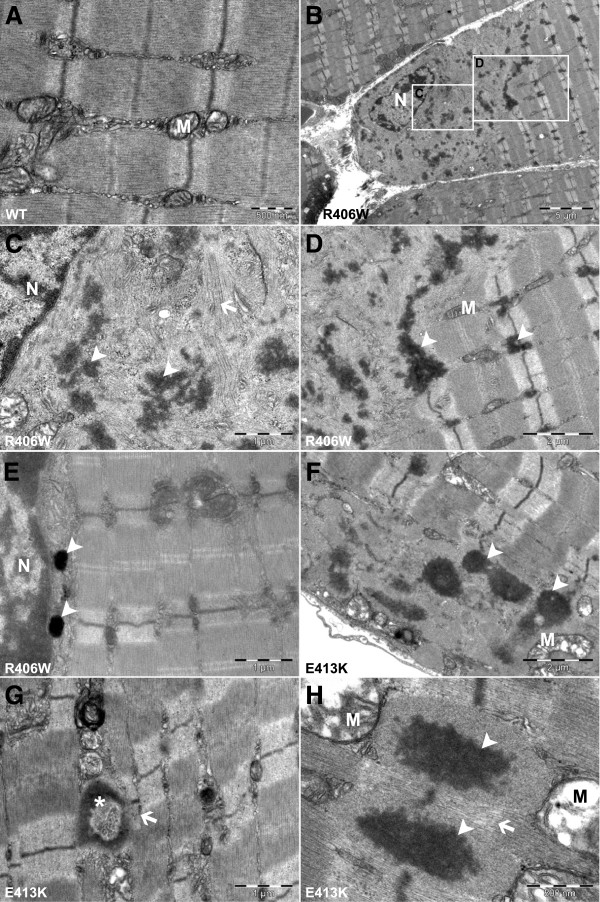
**Ultrastructural perturbations induced by desmin mutants in tibialis anterior muscles.** (**A**) WT, (**B** to **E**) R406W desmin, (**F** to **H**) E413K desmin; expressing tibialis anterior muscles, 1 week (**E**) or 1 month (**A** to **D**, **F** to **H**) after AAV vectors injection. Note that expression of WT desmin does not seem to modify muscle ultrastructure (**A**), whereas expression of R406W mutant of desmin induces the appearance of granulofilamentous electron-dense material (arrowheads, **B** to **E**) located at the perinuclear regions (**B** to **C**), between the intermyofibrillar space and at the Z-line (D); and perturbs mitochondria localization (**D**). Expression of E413K in the tibialis anterior muscle also induces accumulation of granulofilamentous electron-dense material (arrowheads, **F** to **H**) that may form ‘ring-like’ structures (asterisk, **G**). Arrows indicate compressed sarcomere by the accumulation of granulofilamentous electron-dense material. AAV, adeno-associated virus; M, mitochondria; N, nucleus; WT, wild-type.

Using electron microscopy we examined more closely the ultrastructure of the tibialis anterior muscles expressing WT or mutated desmin. All analyses were performed 1 month after injection of AAV vectors, with the exception of R406W expressing muscles for which an additional analysis was also performed 1 week after AAV injection. Muscles expressing WT desmin showed no difference compared to an untreated muscle. Electron microscopy studies disclosed a normal ultrastructure of WT expressing muscles in terms of sarcomere organization, Z-line alignment, distribution and localization of mitochondria, and other organelles (Figure 3A). On the contrary, expression of the R406W desmin mutant resulted in the appearance of granulofilamentous electron-dense material (see arrowheads in Figure 3C,D,E) located in the perinuclear regions (Figure 3B,C) and in the intermyofibrillar space (Figure 3D). Much of this material appeared to be associated with the Z-line and perturbation of the sarcomere organization was observed (Figure 3D). It should be noted that the phenomena was much more pronounced near the nucleus (Figure 3C). In the perinuclear regions, expression of the R406W mutant led to a complete aggregation of the Z-line and severe perturbation of sarcomere alignment, whereas, far away from the nucleus no major alteration of sarcomere organization was detected (Figure 3B,D). As seen in the Figure 3D, accumulation of electron-dense material at the Z-line also perturbed mitochondria localization. Additional ultrastructural analysis showed that accumulation of electron-dense material located in the perinuclear area was already detectable 1 week after AAV-DesR406W injection, but they were not yet located at the Z-line (see arrowheads in Figure 3E). These results emphasize that the R406W desmin aggregates are generated at the perinuclear area and then they spread throughout the whole muscle.

In E413K expressing muscles, 1 month after AAV-DesE413K injection, ultrastructural examination revealed granulofilamentous electron-dense material located under the sarcolemma and between the myofibrils, generally continuous with the Z-lines (see arrowheads in Figure 3F,G,H). Some of these aggregates formed ‘ring-like’ structures of a shape already seen in patients carrying the E413K desmin mutant (see asterisks in Figure 3G). As seen in Figure 3G,H (see arrows), expression of E413K desmin also resulted in compressed sarcomeres with narrower Z-lines, presumably reflecting a loss of myofibril anchorage.

### Impaired muscle function

**Figure 4:**
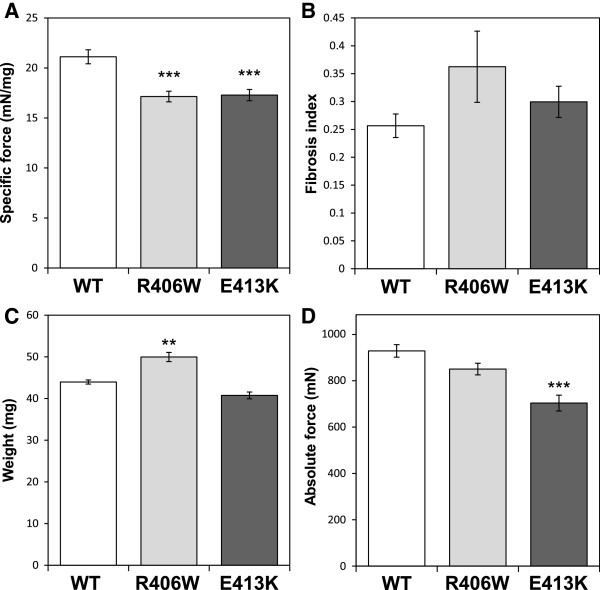
**Altered mechanical characteristics induced by desmin mutants in tibialis anterior muscles.** (**A**) Muscle specific maximal active force, (**B**) muscle fibrosis, (**C**) muscle weight and (**D**) absolute maximal active force were examined in WT (white bars), R406W (light grey bars) and E413K (dark grey bars) desmin expressing tibialis anterior muscles 1 month after AAV vectors injection. Note decrease in muscle specific maximal active force of R406W and E413K expressing muscles compared to WT (**A**). Asterisks indicate a significant difference compared to the WT (*P ≤0.05, **P ≤0.01, ***P ≤0.001). AAV, adeno-associated virus; WT, wild-type.

One of the most marked features of patients with MFM is the reduced maximal active force production of the muscles affected by the disease. A good model of MFM must therefore reproduce this feature. We measured isometric force produced by the tibialis anterior muscles in response to nerve stimulation of mice previously transduced (1 month before) with AAV-DesWT, AAV-DesR406W or AAV-DesE413K. These measures revealed a significant and strong decrease in specific maximal force, that is, maximal force generating capacity, for both R406W (-18.8%, *P* <0.001) and E413K (-18.1%, *P* <0.001) desmin expressing muscles compared to WT (Figure 4A). It should be noted that we did not observe any significant differences in specific maximal force between the muscle expressing WT desmin and untreated muscle (data not shown). The reduced specific maximal force was not related to a significant increase in fibrosis, that is, reduced contractile materials (Figure 4B). We also analyzed the absolute maximal force of a muscle that is directly proportional to its specific maximal active force and weight. The reduction of absolute maximal force only observed for muscles expressing desmin E413K (-24.2%, *P* <0.001) (Figure 4D) was due to a decrease in specific maximal force since it appears that the weight of the muscles expressing the E413K mutant were not significantly lower compared to WT (49.9 mg versus 40.7 mg, *P* = 0.77) (Figure 4D). We also noticed that the increased weight observed for the R406W group (Figure 4C) may be related to the previously observed hyperplasia, as seen in Figure 2B.

## Discussion

The main advantage of the strategy that we have presented is that it is easy to quickly obtain a large number of viral vectors allowing the expression of various desmin mutants and to study the effects of their expression in one genetic background. The idea of creating a disease model by directly expressing a mutant in a given organ has already been performed in rodents for several proteins [34-37]. Thus, by using electroporation, a similar strategy was recently employed by Keduka *et al*., to compare *in vivo* the effects of two mutations of myotilin, a protein involved in MFM [38]. They conclude that this approach recapitulates the pathological changes and the biochemical characteristics observed in patients with myotilin-related MFM. However, the authors have not performed any functional studies and consequently cannot evaluate how their mutants affect muscle physiology. Our results on muscle function reinforce the validity of this strategy since our model reproduces the pathological features of the disease with the major protein responsible for MFM: desmin. Moreover, compared to non-viral transfection techniques such as electroporation, this method significantly improves the percentage of transfected fibers [39,40], is easier to implement *in vivo* and does not induce the confounding effect of muscle damage frequently observed with electroporation. In the present study, we obtained a very homogeneous expression of the mutant desmin with more than 98% of the muscle fibers being transduced and expressing the transgene. AAV-mediated expression of mutated desmin mimics MFM related disorders and seems to be a good model to study molecular heterogeneity of MFM pathogenesis.

Despite the fact that we conducted our study on one muscle (tibialis anterior) at one time point (1 month), we have demonstrated that intramuscular injection of viral vectors to promote the expression of mutant desmin is a potentially useful approach, since the results obtained in this study are very similar to the phenotypes observed in patients. In addition to the loss of muscle specific maximal force, we observe an accumulation of granular aggregates in subsarcolemmal and perinuclear areas of the muscle fibers for the R406W mutation of desmin; while for the E413K mutation, small and dispersed deposits were located both in the center and at the periphery of muscle fibers. These results are very similar to those observed on muscle biopsies of patients [18-20]. In our model we also observe, for the mutation E413K, the typical aggregates with ‘ring-like’ structures are generally continuous with the Z-lines [20]. This approach seems to be adequate and recapitulates extremely well the morphological modifications of muscles in the context of MFM.

Our results also demonstrate that the R406W mutant induces hyperplasia and an increase in muscle mass that is not accompanied by an increase in the absolute maximal active force, as is supposed to be the case for a healthy muscle since specific maximal force is reduced. Each muscle fiber seems to develop less force. This is also true in the case of the E413K mutant. Two main mechanisms may explain this decrease in specific maximal force: a direct disturbance of the generation of force by the sarcomere or an impaired transmission of the force to the tendons. Our observations demonstrate that the sarcomeres are locally disturbed where we observe aggregates of desmin mutants, suggesting that mutations of desmin, by interfering directly with the Z-line, influence force production of muscle fibers. It is also possible that a disturbance in the alignment of the sarcomeres compared to the longitudinal axis of the muscle (pennation angle) or changes in the extracellular matrix may be partly responsible for the decrease in specific maximal force by affecting the force transmission as observed in desmin null mice [41,42]. These points remain to be clarified to determine how much each mechanism is affected by these mutations of desmin.

Finally, in the case of the R406W expressing muscles, we observed focal muscle regeneration, which was confirmed by the presence of immature muscle fibers expressing the neonatal isoform of MHC. This mutation of desmin also induces a decrease in the overall size of fibers. These phenomena, observed 1 month after injection of the virus in tibialis anterior muscle, support the idea that the R406W mutation induces repeated cycles of de-/regeneration of muscle fibers. During regeneration, lost fibers are replaced by clusters of myotubes, formed by satellite cells, which looked like split fibers and have, therefore, a reduced size [43]. This may partially explain the hyperplasia. However, we emphasize that muscle regeneration is not the sole mechanism responsible for the hyperplasia and impaired distribution of muscle fiber size observed in the muscles expressing R406W, since we also observed small fibers expressing exogenous (c-Myc-positive) desmin. The strong regeneration observed in tibialis anterior expressing the R406W mutant of desmin may affect the long-term regenerative capacities of muscles and could explain the disorders observed in MFM. In contrast, the weaker regeneration of muscles expressing the E413K desmin mutant could explain the longer asymptomatic period observed in patients.

## Conclusions

In this study, we show that AAV-mediated expression of desmin mutants in mouse muscles recapitulate the aggregation features, the contractile function decrease and the morphological change observed in MFM patients. In somewhat more detail, differences in muscle regeneration, distribution of muscle fiber size and muscle force production exist between the R406W and E413K desmin mutants. Above all, our results suggest that the R406W mutant of desmin induced a strong muscle regeneration, which is not the case of the E413K mutant. This difference could be at the origin of phenotypic differences observed in patients. It could be interesting, in the future, to understand how these mutations, which affect the same molecular pattern, can induce the observed differences in regeneration. Taken together, our results confirm that AAV-mediated expression of mutants is a useful method to explore clinical phenotypic heterogeneity of a disease, such as MFM.

## Abbreviations

AAV: Adeno-associated virus; CMV: Cytomegalovirus; DAPI: 4^′^,6-diamidino-2-phenylindole; EDTA: Ethylenediaminetetraacetic acid; EGTA: ethylene glycol tetraacetic acid; Fab fragment: Antigen-binding fragment; HSD: Honestly Significant Difference; IgG: Immunoglobulin G; MFM: Myofibrillar myopathy; MHC: Myosin heavy chain; PBS: Phosphate-buffered saline; PCR: Polymerase chain reaction; SDH: Succinate dehydrogenase; SEM: Standard error of the mean; TEM: Transmission electron microscope; WT: Wild-type.

## Competing interests

The authors declare that they have no competing interests.

## Authors’ contributions

PJ carried out the histological, immunostaining and Western blot experiments and analysis, performed the statistical analysis, participated in the design of this study and drafted the manuscript. OC carried out the preparation, purification of the plasmids and site-directed mutagenesis. CH carried out the production, purification and injection of the AAV vectors. AF carried out the muscle force measurements. PV participated in the design of the study and helped to draft the manuscript. JD and GBB participated in the design of the AAV vectors, titration of viral particles and helped to draft the manuscript. OA designed and coordinated the study, obtained the funding, carried out the electron microscopy experiment, performed *in vivo* experiments and analysis and drafted the manuscript. All authors read and approved the final manuscript.

## Supplementary Material

Additional file 1: Figure S1 Z-lines perturbations induced by aggregation of desmin mutants in tibialis anterior muscles. (A) Merge of (B) immunostaining against c-Myc and (C) immunostaining against α-actinin (left part) or specific staining of actin using phalloidin (right part). The plotted graphics reveal the striation pattern of c-Myc (green), α-actinin or phalloidin (red). All analyses were performed 1 month after intramuscular injection of AAV vectors. Note significant perturbations of Z-line located in the areas where exogenous desmin accumulates. AAV, adeno-associated virus. Click here for file
