# Supplementary figures and images for: Viral-mediated expression of desmin mutants to create mouse models of myofibrillar myopathy
Source: Skelet Muscle. 2013 Feb 20;3:4. doi: 10.1186/2044-5040-3-4 (PMC3599656; doi:10.1186/2044-5040-3-4)

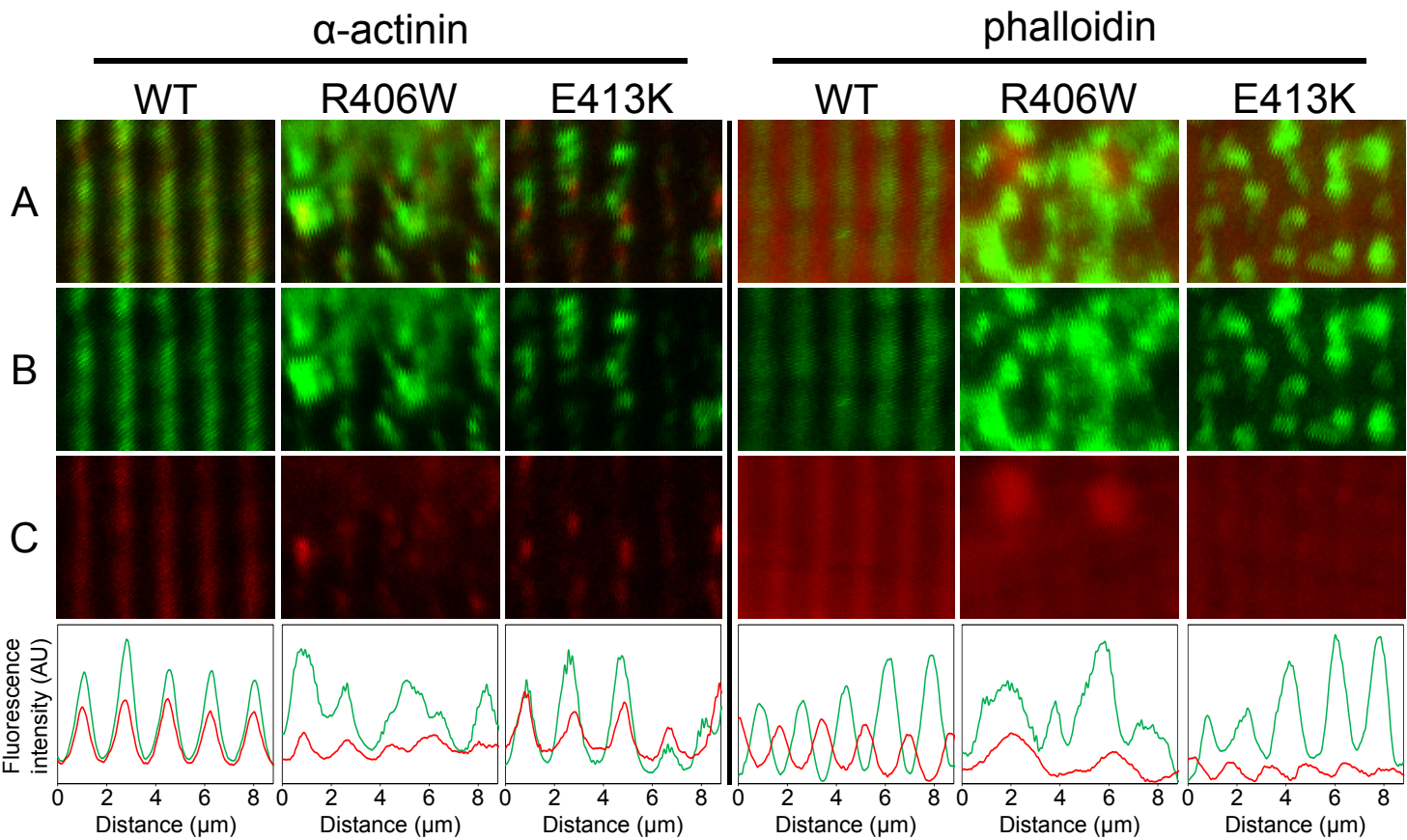

Supplement: Additional file 1: Figure S1 — Z-lines perturbations induced by aggregation of desmin mutants in tibialis anterior muscles. (A) Merge of (B) immunostaining against c-Myc and (C) immunostaining against α-actinin (left part) or specific staining of actin using phalloidin (right part). The plotted graphics reveal the striation pattern of c-Myc (green), α-actinin or phalloidin (red). All analyses were performed 1 month after intramuscular injection of AAV vectors. Note significant perturbations of Z-line located in the areas where exogenous desmin accumulates. AAV, adeno-associated virus. [file 2044-5040-3-4-S1.pdf]
